# Immune profiles in mouse brain and testes infected by Zika virus with variable pathogenicity

**DOI:** 10.3389/fcimb.2022.948980

**Published:** 2022-08-04

**Authors:** Jingzhe Shang, Chunfeng Li, Zhujia Jin, Shulong Zu, Songjie Chen, Junlan Chen, Ziyi Chen, Hua Tang, Cheng-Feng Qin, Qing Ye, Aiping Wu

**Affiliations:** ^1^ Institute of Systems Medicine, Chinese Academy of Medical Sciences & Peking Union Medical College, Beijing, China; ^2^ Suzhou Institute of Systems Medicine, Suzhou, China; ^3^ State Key Laboratory of Pathogen and Biosecurity, Beijing Institute of Microbiology and Epidemiology, Academy of Military Medical Sciences, Beijing, China; ^4^ Institute for Immunity, Transplantation and Infection, School of Medicine, Stanford University, Stanford, CA, United States; ^5^ Center for Systems Medicine, Institute of Basic Medical Sciences, Chinese Academy of Medical Sciences and Peking Union Medical College, Beijing, China; ^6^ Departments of Genetics, School of Medicine, Stanford University, Stanford, CA, United States; ^7^ State Key Laboratory Breeding Base of Basic Science of Stomatology, Ministry of Education, Hospital of Stomatology, Faculty of Medical Sciences, Wuhan University, Wuhan, China; ^8^ Institute of Immunology, Shandong First Medical University, Tai’an, China

**Keywords:** immune profiles, ZIKV, variable pathogenicity, transcriptome, multi-organization

## Abstract

The Zika virus is responsible for neurological diseases such as microcephaly, Guillain-Barré syndrome, neuropathy, and myelitis in human adults and children. Previous studies have shown that the Zika virus can infect nerve progenitor cells and interfere with neural development. However, it is unclear how the immune system responds to infection with Zika viruses with variable pathogenicity. Here, we used two Zika strains with relatively different pathogenicity, the Asian ancestral strain CAM/2010 and the America pandemic strain GZ01/2016, to infect the brains of mice. We found that both strains elicited a strong immune response. Notably, the strain with relatively high pathogenicity, GZ01/2016, caused more intense immune regulation, with stronger CD8+ T cell and macrophage activation at 14 days post infection (dpi), as well as a greater immune gene disturbance. Notably, several TNF family genes were upregulated at 14 dpi, including Tnfrsf9, Tnfsf13, Tnfrsf8, Cd40, and Tnfsf10. It was notable that GZ01/2016 could maintain the survival of nerve cells at 7dpi but caused neurological disorders at 14dpi. These results indicate that Zika viruses with high pathogenicity may induce sustained activation of the immune system leading to nerve tissue damage.

## Introduction

Zika virus (ZIKV) is a single-stranded RNA mosquito Flaviviridae virus. Recently, ZIKV infection has become a global health problem (WHO, 2018). Since the first outbreak in Yapo Island in 2007, ZIKV continues to spread from the South Pacific Islands to the American continent (Gatherer and Kohl, 2016). ZIKV has caused widespread concern in international communities because of its association with microcephaly in infants (Mlakar et al., 2016).

ZIKV affects the development of neural progenitor cells and peripheral neurons, leading to microcephaly (Dang et al., 2016; Li et al., 2016; Tang et al., 2016; Oh et al., 2017). However, ZIKV also causes immune-mediated disorders in adults and adolescents, including Guillain-Barre syndrome (GBS), neurodegenerative diseases, and multiple sclerosis (Oehler et al., 2014; Araujo et al., 2016; Cao-Lormeau et al., 2016; Petersen et al., 2016; Alves-Leon et al., 2019). In an epidemic study in Brazil, some adults with ZIKV infection developed GBS (Ansar and Valadi, 2015). Since the first case of GBS associated with ZIKV was reported in 2013 in French Polynesia, new cases have increased significantly (Oehler et al., 2014; Cao-Lormeau et al., 2016).

ZIKV infection induces an inflammatory response in the brain. CD8^+^ T cells play a key role in the resistance to ZIKV infection. Animal studies have shown that the decrease of CD8^+^ T cells increased the amount of virus, while the deletion of CD8^+^ T cells resulted in the death of mice (Ngono et al., 2017). Recent studies have found that ZIKV specific CD8^+^ T cells have multiple functions and antiviral activities (Grifoni et al., 2018). Analysis of infected blood samples revealed that ZIKV of African and Asian strains mainly infects CD14^+^ monocytes in blood (Foo et al., 2017). In mice, ZIKV infection increases the incidence of epilepsy. Moreover, ZIKV-induced neuroinflammation is alleviated when TNF-α is inhibited (de Oliveira Souza et al., 2018). In addition to neuronal cells, ZIKV can infect microglia in the brain and cause inflammation (Lum et al., 2017; Meertens et al., 2017). These studies show that ZIKV infection induces changes in the immune microenvironment of the brain; however, the relationship between the inflammatory response and neurological damage remains unclear.

Another concern with ZIKV infection is its long-term persistence in the testes (Atkinson et al., 2017). A long-term follow-up study showed that ZIKV persists in the semen of infected patients for up to 141 days, which leads to a potential risk of sexual transmission (Mansuy et al., 2016). Further, studies in mice have shown that ZIKV can cause testicular damage and male infertility (Govero et al., 2016; Ma et al., 2016). However, the impact of ZIKV infection on the immune microenvironment in the testes remains unknown

Evolution analysis found that the Asian and American strains of ZIKV that have been responsible for the outbreaks of microcephaly belong to two branches. Earlier studies have shown that ZIKV strains from different sources have different pathogenic effects (Tripathi et al., 2017). Recent experiments have found that the Asian ancestral strain (CAM/2010) does not cause significant neurological diseases, and have reported that a mutation in the perM protein (S139N) of the CAM/2010 led to a significant increase in ZIKV neurovirulence (Yuan et al., 2017).

To further explore the immune response of the host to different virulent strains of ZIKV, we infected early-born mice to study the dynamic immune responses in the brain and testes. We found that both of the tested strains (CAM/2010, GZ01/2016) elicited immune responses in the brain, with GZ01 causing the stronger response. In GZ01 infected mice, activated CD8^+^ T cells were more potent; this process involves multiple Tnfs and Tnf receptors, such as Tnfrsf9, Tnfsf13, and Tnfrsf8. These results indicate that GZ01 may cause continuous inflammation in brain tissue, leading to more severe tissue damage than CAM. Additionally, neither virus caused a significant immune response in the testes through distant infection.

## Materials and methods

### Animals and virus

C57 mice were purchased from the Vital River Laboratory (Beijing, China) and bred in our core animal facility. All animal experiments were performed according to the standard operating protocol (SOP) issued by the Animal Experiment Committee of the Laboratory Animal Center, Academy of Military Medical Sciences, China (IACUC-13-2016-001). ZIKV strains GZ01/2016 (GZ01, GenBank: KU820898) and CAM/2010 (CAM, GenBank: JN860885) were used according to the methods mentioned previously (Li et al., 2017).

### ZIKV infection

Eight-day-old neonatal mice were intracerebrally injected with 100 PFU of the virus, the brain and the testes were collected at 7, 14, and 33 days after infection to analyze the immune response.

### Flow cytometry and cell sorting

For brain single cell preparation, mice were anesthetized and received cardiac perfusion with 20 ml 0.01 M phosphate buffered saline (PBS). For cell surface staining, prepared single cell suspensions were first blocked with anti-Fcγ III/II Receptor mAb (2.4 G2) for 5 min, followed by staining with fluorescence-conjugated mAb for CD45.2 (104), CD3ϵ (145-2C11), NK1.1 (PK136), CD4 (GK1.5), CD8 (53-6.7), CD11c (N418), IA/IE (M5/144.15.2), CD64 (X54-5/7.1.1), F4/80 (BM8), CD11b (M1/70), and Ly6C (HK1.4). All mAbs were purchased from eBioscience, except CD3, CD64, CD11b mAb (BD), and CD4, IA/IE mAb (Biolegend).

### RNA isolation and sequencing

We fed 1 μg total RNA from mice sample into the NEBNext PolyA mRNA Magnetic Isolation Kit (NEB, catalog #E7490L), then constructed the specific chain RNA library using the NEBNext Ultra Directional RNA Library Prep Kit for Illumina (NEB, catalog #E7420L). We performed library construction according to the vendor’s instructions, starting with the chapter “Protocol for use with NEBNext Poly (A) mRNA Magnetic Isolation Module.” The mRNA is enriched by magnetic beads, followed by first- and second-strand cDNA synthesis. Double-stranded cDNA was purified using Agencourt AMPure XP Beads for cDNA library construction. The quality of the library was evaluated on the Agilent 2100 bioanalyzer and quantified by qPCR using the VAHTS library quantification Kit for Illumina (Low ROX Premixed) (Vazyme, catalog #NQ103). Libraries were sequenced on the HiSeq X10 using the paired end 2*150bp, single-index format. The RNA-seq data were deposited in the National Center for Biotechnology Information (NCBI) under accession code PRJNA759363 and PRJNA835059.

### Mass spectrometry

LC-MS based tandem mass tag (TMT)-labeled identification and quantification of the proteome were applied for the proteomics profiling of tissue samples. In brief, tissues were lysed in a fresh prepared lysis buffer containing 6 M Gdmcl, 10 mM TCEP, 40 mM CAA, and 500 mM Tris (pH 8.5). After protein reduction and alkylation, the protein concentration was measured using the BCA kit (ThermoFisher). Next a 100-μg protein pellet was used for trypsin digestion (1:50) overnight at 37°C. Peptides were cleaned using a Waters HLB column. Samples were randomized and labeled using TMT10 Plex (ThermoFisher) in 100 mM TEAB buffer. An equal number of peptides from each sample were pooled together as a reference. Data acquisition was performed on a NanoAquity 2D nanoLC (Waters) directly coupled in-line with a linear trap quadrupole (LTQ)-Orbitrap Velos instrument (Thermo Scientific) *via* a Thermo nanoelectrospray source. Subsequently, 3 μg of multiplexed sample was loaded on LC-MS analysis (15 μg multiplexed sample was loaded on 2D-LC for online fractionation). Peptides were separated by reverse-phase chromatography at high pH in the first dimension, followed by an orthogonal separation at low pH in the second dimension. The raw data were processed with the Proteome Discoverer 2.1 (Thermo), using the UniProtKB database, applying 1% FDR. Missing values were imputed with a random number below 50% of the limit of detection. The proteome data were deposited in the integrated proteime resources (iProX) under accession code IPX0004641000.

### Bioinformatics

Raw transcriptome data of the mouse brain were obtained from the NCBI (PRJNA759363). We used a unified process to produce the raw Fastq data of mouse brain and testes. The quality of the Fastq data was verified by FastQC software. Connectors, primers, and low-quality reads were removed by Trimmomatic. Clean reads were compared to the musculus. G38.p5 genome using the STAR software. Finally, the expression of each gene was quantified by RSEM, and DESeq2 software was used for data standardization and differential gene analysis. The screening criteria of differentially expressed genes (DEGs) were adjusted p < 0.05 and fold change > 1.5. Then, clusterProfiler was used for GO enrichment analysis.

### Immune cell type identity

The ImmuCC website provides an analysis of the percentage of immune cells in mouse samples. The whole tissue was used as a mixed model of different immune cells. Based on the immune cell characteristic gene set, the proportion of different immune cells was calculated using the support vector regression (SVR) algorithm. The proportion of ten immune cells added up to 100. To analyze the subtypes of CD8^+^ T cells and macrophages, we first collected the related gene sets from the article and used the ssGSEA method to calculate their over representation in tissues.

### Gene set enrichment analysis

To analyze the different functions of immune cells, we collected the gene sets of different functional modules from IPA. The genes in each comparison group were sorted by fold change. The correlation is represented by normalized enrichment score (NES)

### Module score

We calculated the disease gene module score of each sample using the Gene Set Variation Analysis (GSVA) method (Hänzelmann et al., 2013). GSVA first determines the high or low expression of a gene in the sample, then sorts and standardizes the genes in each sample. Finally, the ES score is calculated using the Kolmogorov–Smirnov (KS) like random walk statistic.

### Interaction network

We obtained the target gene interaction network through the commercial software IPA. The reference database removes the third-party database and only the IPA knowledge base verified by experts is selected. The network diagram was generated using Cytoscape software.

## Results

### Similarities and differences between GZ01 and CAM infected mice

**Figure 1:**
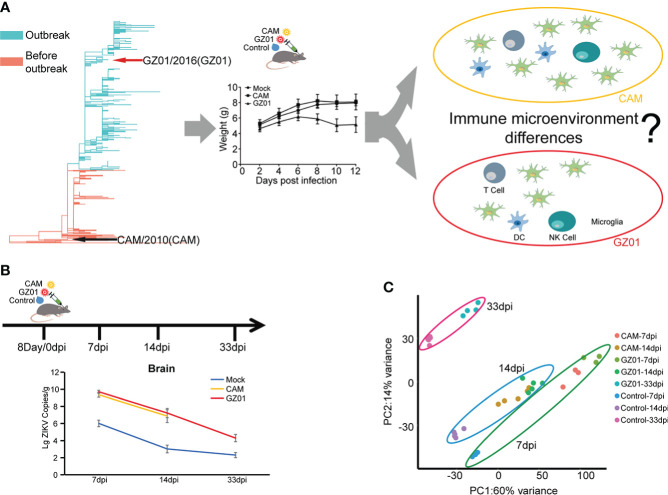
Schematics for identifying immune signatures from ZIKV-infected mouse brains. **(A)**, Different immune response of mice caused by different strains of Zika virus. Two representative ZIKV strains, CAM and GZ01, were selected and are indicated with black and red arrows in the phylogenetic tree. **(B)**, Experimental proposal for building a mouse model and data analysis. Mice were inoculated with 100 pfu of ZIKV strains or control medium on the eighth day after birth. Brain samples were collected at 7, 14, and 33 dpi for further experiments. Viral loads of ZIKV in mouse brains are shown in line chart. **(C)**, PCA displaying biological variation among different infection groups.

The outbreak of ZIKV in Brazil caused a global panic due to its association with microcephaly. However, the ZIKV identified earlier in Asia did not appear to cause microcephaly (Yuan et al., 2017). Phylogenetics showed that two strains have a distant evolutionary relationship (Yuan et al., 2017). After the mice were infected with two representative strains (GZ01 and CAM), the GZ01 strain more significantly reduced the weight of the mice. However, the difference in the disturbance of the immune microenvironment caused by the two viruses remained unclear (**Figure 1A**). To answer this question, we chose GZ01 and CAM strains to study the immune signature changes in mice. On the basis of previous studies, we found that mice had significant changes at 14 days post infection (dpi; infection being the eighth day after birth). So, we collected 7 dpi, 14 dpi, and 33 dpi samples to examine the changes in immune signatures over time (**Figure 1B**). ZIKV RNA copies were both increased and no significant difference in ZIKV-infected mice were observed until 14 dpi. (**Figure 1B**). We detected differences between samples by principal component analysis (PCA) and found that ZIKV-infected mice had both similarities and differences to control mice (**Figure 1C**). The first two principal components represented a 74% change of 6146 genes. PC1 is associated with infection strains, while PC2 is associated with infected time.

### Transcription disturbances in the GZ01- and CAM-infected mice brains

The coordinated expression of genes may be related to specific biological functions, a modular analysis method was used to clarify the biological functions of different virulence strains. We clustered 6146 perturbation genes into 31 modules by k-means strategy. The genes in each module had similar expression patterns. The biological functions of each module were annotated through the Metascape website (**Supplementary Table 1**). The score for each module was calculated using Chaussabel’s method (Chaussabel et al., 2008). Pie chart is used to show the proportion of DEGs in each module.

**Figure 2:**
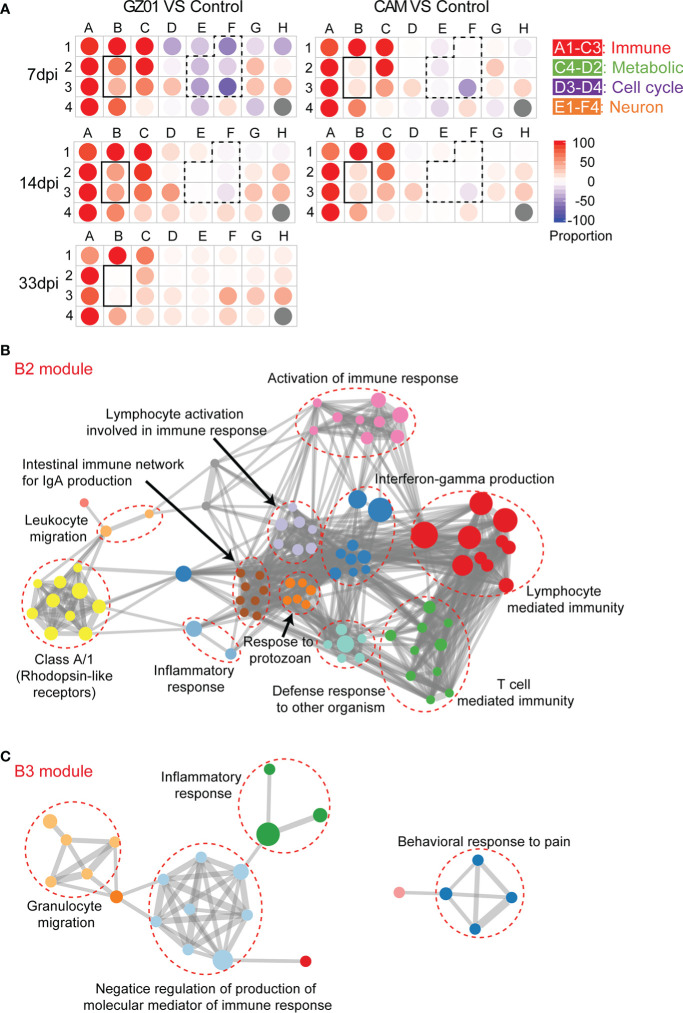
Identify key difference modules between CAM and GZ01 infected mice. **(A)**, Modular map analysis: Gene expression from mice infected with CAM and GZ01 and Control mice were compared (P < 0.05, Fold change > 1.5). Spots indicate the proportion of genes significantly that were changed for each module in mice infected with CAM and GZ01 compared to controls. Red: Overexpressed, Blue: Underexpressed. (**B, C**), Network of enriched terms of the B2 and B3 modules, colored by cluster ID, where nodes that share the same cluster ID are typically close to each other.

The annotated modules are arranged according to biological function, the A1-C3 module is related to the immune response, and E1-F4 is related to neural development (**Figure 2A**, **Supplementary Table 1**). Based on a comprehensive comparative analysis, two unique patterns were identified. One pattern is related to the immune response; most immune modules increased in both groups of mice, whereas B2 and B3 were more increased in GZ01-infected mice than in CAM-infected mice. Another pattern is associated with neural development; multiple neurological modules decreased in GZ01-infected mice compared to CAM-infected mice. To further capture the relationships between the terms, a subset of enriched terms was selected and rendered as a network plot. The network of the B2 module revealed that the enriched pathways enhanced in GZ01 were linked to Activate immune response, Interferon-gamma production, Lymphocyte-mediated immunity, and T cell-mediated immunity (**Figure 2B**). The B3 module was enriched for the inflammatory response, granulocyte migration, and negative regulation of the production of molecular mediators of the immune response (**Figure 2C**). These results suggest that the GZ01 strain induces a stronger immune response involving T cell activation and interferon production compared to the CAM strain in mouse brains.

### Immune cell profiles of the brain in GZ01- and CAM-infected mice

**Figure 3:**
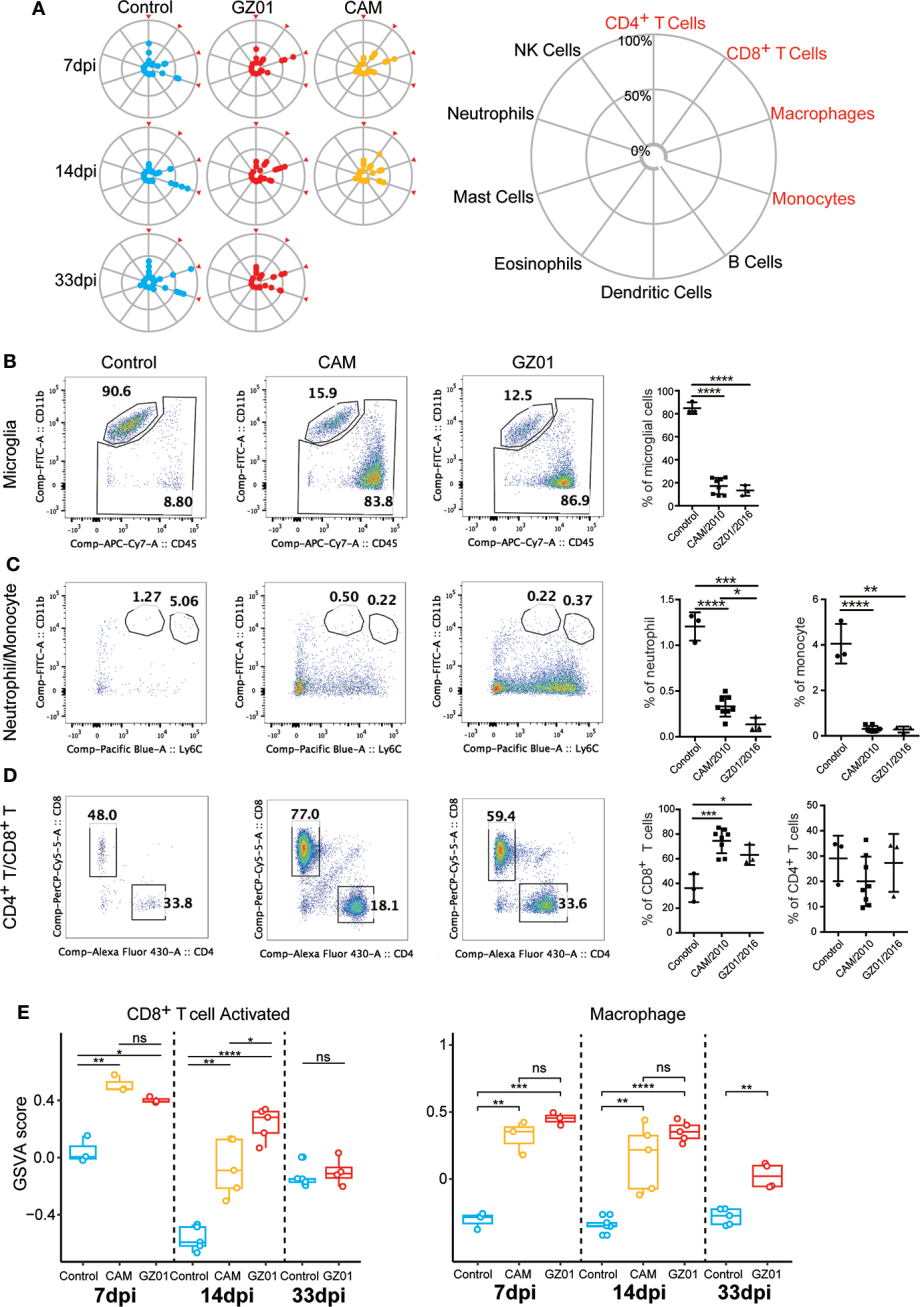
Immune cell profiles of mice infected with ZIKV and Control. **(A)**, Zika infection leads to activation of CD8+ T and microglia cells. A, The proportions of the ten immune cell types investigated. The immune cell ratio was calculated using expression data from the immuCC website. **(B–D)**, Flow cytometry immunophenotypic analysis of a case of microglia, monocyte, CD4, and CD8 in Control and mice infected with ZIKV. **(E)**, Comparison of the enrichment scores of immune cell subtypes in different infection groups by GSVA.*P < 0.05; **P < 0.01, ***P < 0.001, ****P < 0.0001, ns, not significant (two-tailed unpaired Student’s t-test).

We first examined the changes in the composition of immune cells in all samples. The proportions of ten types of immune cells were detected, of which four types of immune cells (CD8^+^ T cells, CD4^+^ T cells, macrophages, and monocytes) accounted for the majority (**Figure 3A**). We used flow cytometry to analyze the true ratios of the four types of immune cells. The results showed that the proportion of microglia and monocytes in ZIKV-infected mice was significantly lower than that in control mice (**Figures 3B, C**). However, the proportion of CD8^+^ T cells was significantly higher than that in control mice (**Figure 3D**). Additionally, the proportion of CD4^+^ T cells was not significantly different among the three experiments (**Figure 3D**).

As immune cells have multiple states, we further analyzed the changes in the functional sets of CD8^+^ T cells and macrophages. The CD8^+^ T cells in the GZ01 infected mice were activated for longer, and the CD8^+^ activation function of the CAM-infected mice was significantly lower than that of the GZ01-infected mice at 14 dpi (**Figure 3E**). Moreover, the function of macrophages was significantly stronger in both strains compared to the Control, and there was no significant difference between the two strains at 7 dpi and 14 dpi.

### Immune molecular perturbation between GZ01- and CAM-infected mice

**Figure 4:**
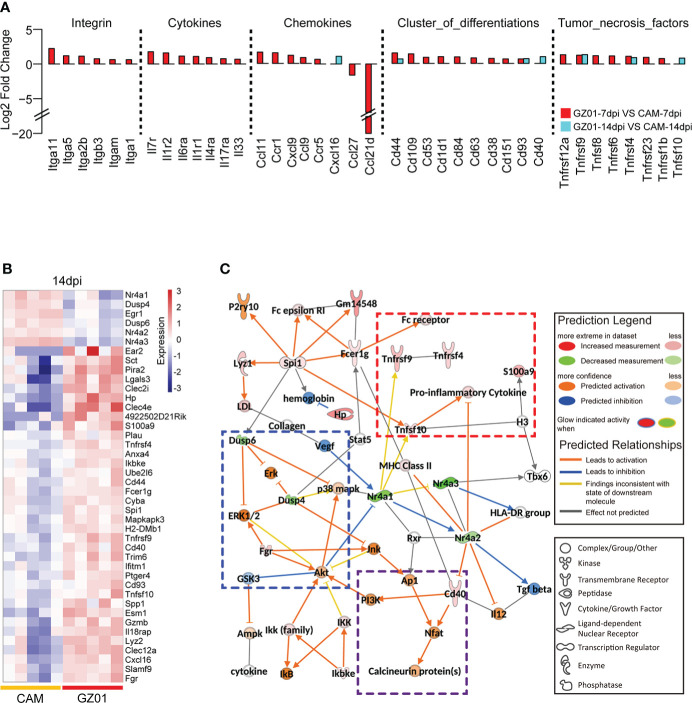
Differential expression profiles of immune molecules between two strains infected mice. **(A)**, Fold change of differentially expressed cytokines between mice infected with GZ01 and CAM at 7 dpi and 14 dpi. **(B)**, Heatmap of differentially expressed immune genes between CAM and GZ01 at 14 dpi. **(C)**, Regulatory networks of differentially expressed immune genes and predictive regulatory genes at 14 dpi.

We next examined the perturbation of immune response molecules in ZIKV-infected mice. To this end, we collected a set of immune response molecules, which obtained 3335 direct and indirect immune response genes. Using a cut threshold of fold change > 1.5 and padj < 0.05, differentially expressed immune response genes were identified between GZ01- and CAM-infected mice. At 7 dpi, multiple cytokines were significantly upregulated in GZ01-infected mice compared to CAM-infected mice. However, at 14 dpi, only seven cytokines were significantly upregulated, including Cxcl16, Cd44, Cd93, Cd40, Tnfrsf9, Tnfrsf4, and Tnfsf10 (**Figure 4A**).

Moreover, 245 and 42 DEGs were obtained at 7 dpi and 14 dpi, respectively ( **Figure S1A** and **Figure 4B**). IPA software was used to analyze the two gene sets, and both classical pathways and upstream regulatory factors were identified. At 7 dpi, the activation pathway in GZ01-infected mice was found to involve multiple acute inflammatory signals, including acute phase response signaling, the Th17 activation pathway, and TREM1 signaling. Additionally, GZ01-infected mice showed activation of the neuroinflammation signaling pathway (**Figure S1B**). IPA software was used to predict the regulatory network of differential gene sets at 14 dpi; this regulatory network predicts the function of input and related genes. In this regulatory network, three functional sets were observed: pro-inflammatory signal, MAPK activation signal, and T cell signal (**Figure 4C**). The inflammatory signals included Tnfsf10, Tnfrsf9, Tnfrsf4, and S100a9. Tnfrsf9 involves the development, survival, and activation of T cells (Miller et al., 2002), while Tnfrsf4 is a co-stimulator of long-term T cell immunity (Hendriks et al., 2005). The activation of the MAPK pathway was predicted on the basis of the down-regulation of Dusp4/6. Cd40 binds to the Nfat gene to induce secretion of cytokines by T cells (Munroe and Bishop, 2007).

Immune checkpoints play an important role in the immune regulation process. We compared the expression differences between immunoinhibitor- and immunostimulator-related genes in different samples. The results showed that both strains significantly upregulated immunosuppressives and immunostimulatory genes relative to the control mouse brain (**Figure S2A**). Further, DEGs from the immunostimulatory gene set showed that GZ01-infected mice had a higher FC than CAM-infected mice. Notably four Tnf factors were specifically upregulated in GZ01-infected mice at 14dpi, including Tnfrsf9, Tnfsf13, Tnfrsf8, and TNfrsf5 (**Figures S2B, C**).

### Nervous system function is significantly influenced by the GZ01 strain compared to the CAM strain

**Figure 5:**
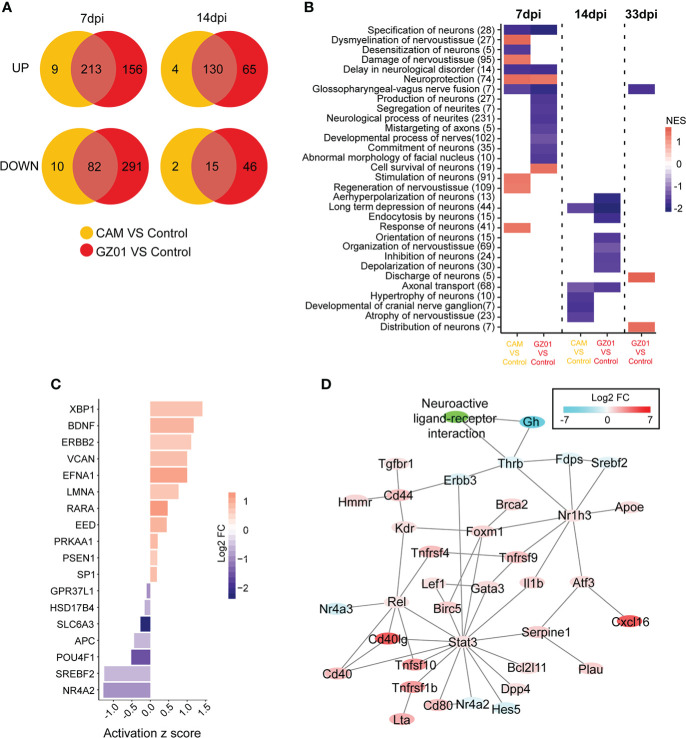
Characteristics of neurological disturbance in different infection groups. **(A)**, The common and specific gene numbers between GZ01 and CAM infections are shown in the Venn diagram. **(B)**, GSEA scores of neurological gene set in different comparison groups. **(C)**, Upstream regulatory factors for GZ01-specific genes related to neurological function at 7 dpi. **(D)**, Regulatory network based on GZ01-specific differentially expressed neural genes at 14 dpi.

After collecting multiple neurological disease gene sets from IPA to analyze the effects of different strains on the nervous system, we first integrated all nerve function genes into a single gene set. A Venn plot showed that there were fewer specific DEGs in CAM-infected mice, but more specific DEGs in GZ01-infected mice (**Figure 5A**). Next, we examined the difference in neurological function between different strains of infection. At 7 dpi, CAM-infected mice developed neuronal damage and demyelination of nervous tissue, and simultaneously, activated neuronal stimulation and regeneration. However, GZ01-infected mice showed specific down-regulation of multiple neural developmental functions and increased neurological survival. At 14 dpi, atrophy of nervous tissue was down-regulated in CAM infected mice. In GZ01-infected mice, the function set of multiple nerve signals were down-regulated (**Figure 5B**). These results suggest that GZ01 and CAM have varying effects on neurological function in mice. At 7 dpi, the neurological development of GZ01-infected mice was affected, whereas the neurological function of CAM-infected mice had begun to recover. By 14 dpi, the nerves of GZ01-infected mice had been damaged, resulting in weakened nerve signal function.

To further identify the most important neuroregulatory genes, we analyzed the neuro-related genes with specific changes in viral strains. For neuron-associated genes with specific changes in GZ01-infected mice at 7 dpi (156 upregulated genes, 291 down-regulated genes), most upstream regulators of GZ01-specific disturbances have been shown to be related to neural development (**Figure 5C**), including Efna1, Lmna, Nr4a2 (Jakaria et al., 2019), Pou4f1 (Zou et al., 2012), and Slc6a3 (Arpón et al., 2018). Additionally, Bdnf is related to nerve survival and disease (Hu and Russek, 2008). At 14 dpi, the network of GZ01 strain-disturbed genes can be divided into two parts (**Figure 5D**): One part constitutes an immune molecular network with the STAT3 gene as the core (most of these genes have upregulated expression), and another part related to nerve signal, including Thrb, Gh, and other down-regulated genes.

### ZIKV infection induces upregulation of apoptosis-related proteins at 14 dpi

**Figure 6:**
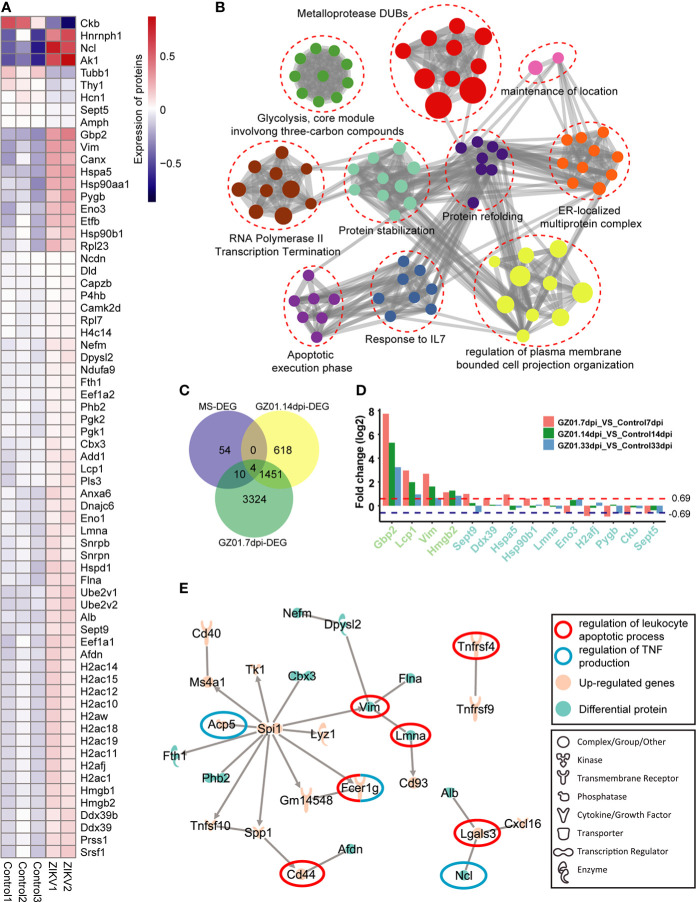
Proteomics profiles of mice infected with GZ01 at 14 dpi. **(A)**, Differentially expressed proteins in GZ01 infection mice relative to Control. **(B)**, Network of enriched terms of differential proteins, colored by cluster ID, where nodes that share the same cluster ID are typically close to each other. **(C)**, The overlapping gene numbers between DEGs of proteomics and DEGs of transcriptome. **(D)**, Fold change of the overlapping genes between mice infected with ZIKV and control. The lime-green label indicates the four overlapping genes between proteomic data and transcriptional data at 14 dpi. The cyan label indicates the ten overlap genes between proteomic data and transcriptional data at 7 dpi. **(E)**, Regulatory network between differential proteins and the upregulated genes in transcriptome data.

The transcriptome data is insufficient to provide a picture of the correct biological state, particularly regulatory processes and post-translational modifications. The proteomic data of ZIKV-infected mice at 14 dpi were obtained by mass spectrometry, and we observed 68 differentially expressed proteins between infected and control mice (**Figure 6A** and **Supplementary Table 2**). The immune signals involved in these proteins include the apoptotic execution phase and response to IL-7 (**Figure 6B**), a cytokine that is important for B and T cell development (ElKassar and Gress, 2010). We found 14 genes that overlapped with the differential genes from transcriptome, including 10 differential genes in the 7dpi sample and 4 differential genes in the 14dpi sample (**Figure 6C**). Among them, 3 genes (Gpb2, Lcp1, and Vim) are significantly up-regulated at 7dpi in GZ01 infected mice against control mice (**Figure 6D**). The relationship between the differential proteins and the differential genes is worth exploring. Based on the genes in the two groups, we predicted the regulatory network between the transcriptome and the Proteome (**Figure 6E**). We found that two hub genes, Spi1 and Vim, are important bridges between the transcriptome and Proteome.

### Immune signal disturbances in testicular tissue are weak

Studies have shown that ZIKV exists for a long time in testicular tissues. As the stimulated distal tissue, we detected the disturbance of its immune signal. We found that the virus in the testes of ZIKV-infected mice was undetectable from 7 dpi (**Figure S3A**). The PCA results showed that at the same time point, the difference between different infection mice was small (**Figure S3B**). There was no significant difference in the proportion of immune cells between different infection groups (**Figure S3C, D**), nor was there a significant difference in CD8^+^ T cell function. However, the function of macrophages in the CAM-infected mice was stronger than that in the GZ01 infected mice at 7 dpi (**Figure S3E**). The immune stimulation and immunosuppressive functions also showed no significant change in either of the infected testicular tissues (**Figure S2D**).

## Discussion

After preliminary experiments, we found that the mouse phenotype changed significantly at 14 dpi after infection with ZIKV, including observable weight loss (**Figure 1A**). There was no significant difference in the viral load of mice infected with different strains, showing that the virus replication ability of different virulence strains was similar. Therefore, this time point can be considered suitable for detecting the immune response mechanism against ZIKV infection. We selected the Asian ancestral strain CAM and the intensity strain GZ01 from South America and China for a comparative study to explore the differences in immune profiles. Further, we explored the relationship between the immune response and nerve injury.

Based on the gene expression module, we found that ZIKV infection induces immune responses and affects neural development in the mouse brain. For the immune response, ZIKV activates ISG antiviral signaling and induces an acute response, typically activation of NK-κb signaling and interferon production (**supplementary Table 1**), which is consistent with the study of Zhang et al., who also found that GZ01 can more strongly induce the expression of TNFα and IFNα (Zhang et al., 2017). Several cytokines were upregulated at 7 dpi between GZ01 and CAM infected mice, expect for Tnfrsf9, Tnfsf13, Tnfrsf8, and Entpd1, these genes were upregulated in GZ01 infected mice at 14dpi (**Figure 4A**). Tnfrsf9 is known to stimulate T cells and induce strong anti-cancer and anti-autoimmune effects (Ye et al., 2002; Vinay et al., 2006). Tnfsf13 is believed to attenuate influenza virus replication in later stages (Tran et al., 2013). Tnfrsf8 is expressed in activated T cells and B cells, which activate NF-κb signaling (Higuchi et al., 2005). Furthermore, upregulation of Tnfrsf8 is linked to patients with chronic inflammatory diseases (Oflazoglu et al., 2009). These results show that GZ01 infection causes chronic inflammation in the mouse brain.

GZ01-infected mice showed a stronger immune response than CAM-infected mice at 7 dpi. Although CD8^+^ T cell activation function was stronger in both ZIKV infected mice than in control mice at 7 dpi, CD8^+^ T cell activation was significantly stronger in GZ01-infected mice than in CAM-infected mice at 14 dpi. These results indicate that GZ01 continues to activate CD8^+^ T cells at later stages.

At the level of immune molecules, several immune genes were upregulated in GZ01-infected mice, which were related to proinflammatory activity, MAPK activation, and T cell activation. The GZ01 strain also specifically upregulates the expression of Cxcl16, Cd40, and Tnfsf10 at 14 dpi. Cxcl16 acts as a scavenger in macrophages and has a strong chemotactic effect (Van Der Voort et al., 2005), Cd40 is important for inflammatory responses including T cell mediated immunity and memory B cell development (van Kooten and Banchereau, 1997; Bennett et al., 1998), and Tnfsf10 induces apoptosis (Kuribayashi et al., 2008). The proteomic data also showed that the GZ01 strain enhanced apoptosis signals. Vim and Lmna proteins were upregulated in GZ01 infected mice. Lmna play a key role in nuclear envelope integrity maintenance and nuclear architecture (Cohen et al., 2001).

To explore the relationship between the immune response and nerve damage, we compared the effects of different strains on neurological function. We observed different regulatory patterns between CAM- and GZ01-infected mice at 7dpi. Nerve damage and nerve regeneration were significantly enhanced in CAM-infected mice, while in the GZ01-infected mice, the nerve survival ability was enhanced and multiple neurodevelopmental functions were decreased. Thus, the GZ01 strain appears to be more potent in suppressing the immune system and enhances the survival of nerve cells to complete proliferation in infected cells. The difference between the two strains was more pronounced at 14 dpi. The neurotrophic function was reduced in the CAM-infected mice, whereas the multiple neurodevelopmental functions in the GZ01-infected group were still low. These results suggest that the greater virulence of GZ01 may be associated with a stronger, sustained immune response, which in turn leads to brain tissue entering a chronic inflammatory state at 14 dpi.

In summary, we compared the effects of different ZIKV strains on the immune system of mouse brain and found that GZ01 infected mice had a longer lasting inflammatory response compared with CAM strain. GZ01 inhibited the neuronal apoptosis signal and replicated continuously in the infected cells. As a result, the immune system seems continuously activate by the Tnf signal, which affects the development of brain tissue and nerves. However, this hypothesis still needs further experimental verification.

## Data availability statement

The datasets presented in this study can be found in online repositories. The names of the repository/repositories and accession number(s) can be found below: The RNA-seq data were deposited in the National Center for Biotechnology Information (NCBI) under accession code PRJNA759363 and PRJNA835059. The proteome data were deposited in the integrated proteime175 resources (iProX) under accession code IPX0004641000.

## Ethics statement

The animal study was reviewed and approved by Institutional Animal Care and Use Committee. Written informed consent was obtained from the owners for the participation of their animals in this study.

## Author contributions

Conceptualization, AW, and CL. Methodology, JS, CL, SZ, SC, JC, ZC, and HT. Validation, AW, JS, and CL. Formal analysis, JS, CL, ZJ, SC, and HT. Investigation, AW, CL, JS, and ZJ. Data curation, JS, and ZJ. Writing—original draft, JS, AW, and CL. Writing review and editing, JS, AW, CL, C-FQ and QY. Visualization, JS, and CL. Supervision, JS, and AW. Funding Acquisition, JS, and AW. All authors contributed to the article and approved the submitted version.

## Funding

This work was supported by the National key research and development program (2021YFC2301300); the CAMS Innovation Fund for Medical Sciences (2021-I2M-1-061); the National Natural Science Foundation of China (92169106, 31900472); the special research fund for central universities, Peking Union Medical College (2021-PT180-001); Suzhou science and technology development plan (szs2020311).

## Conflict of interest

The authors declare that the research was conducted in the absence of any commercial or financial relationships that could be construed as a potential conflict of interest.

## Publisher’s note

All claims expressed in this article are solely those of the authors and do not necessarily represent those of their affiliated organizations, or those of the publisher, the editors and the reviewers. Any product that may be evaluated in this article, or claim that may be made by its manufacturer, is not guaranteed or endorsed by the publisher.

## Supplementary material

Supplementary Figure 1
**(A)**, Heatmap of differentially expressed immune genes between CAM and GZ01 at 7 dpi. **(B)**, Functional enrichment of differentially expressed genes between CAM and GZ01 at 7 dpi.

Supplementary Figure 2 Evaluation of immune checkpoint in tissues from mice infected by each strain. **(A)**, Calculation of the immune checkpoint scores of mouse brain by GSVA. **(B, C)**, Differential fold changes of immune stimulation gene from brain at 7 dpi and 14 dpi. **(D)**, Calculation of immune checkpoint scores of mouse testes by GSVA. *P < 0.05; **P < 0.01, ***P < 0.001, ****P < 0.0001, ns, not significant (two-tailed unpaired Student’s t-test).

Supplementary Figure 3 Changes in the immune microenvironment of mice testes. **(A)**, Viral loads of ZIKV in the mouse shown in line chart. **(B)**, PCA displaying biological variation among different infection groups. **(C)**, The proportion of ten main immune cell types. The immune cell ratio was calculated using expression data from the immuCC website. **(D)**, Comparison of the immune cell ratio of immune cell subtypes in different infection groups. **(E)**, Comparison of the enrichment scores of immune cell subtypes in different infection groups by GSVA.
