# Supplementary figures and images for: Immune profiles in mouse brain and testes infected by Zika virus with variable pathogenicity
Source: Front Cell Infect Microbiol. 2022 Aug 4;12:948980. doi: 10.3389/fcimb.2022.948980 (PMC9385972; doi:10.3389/fcimb.2022.948980)

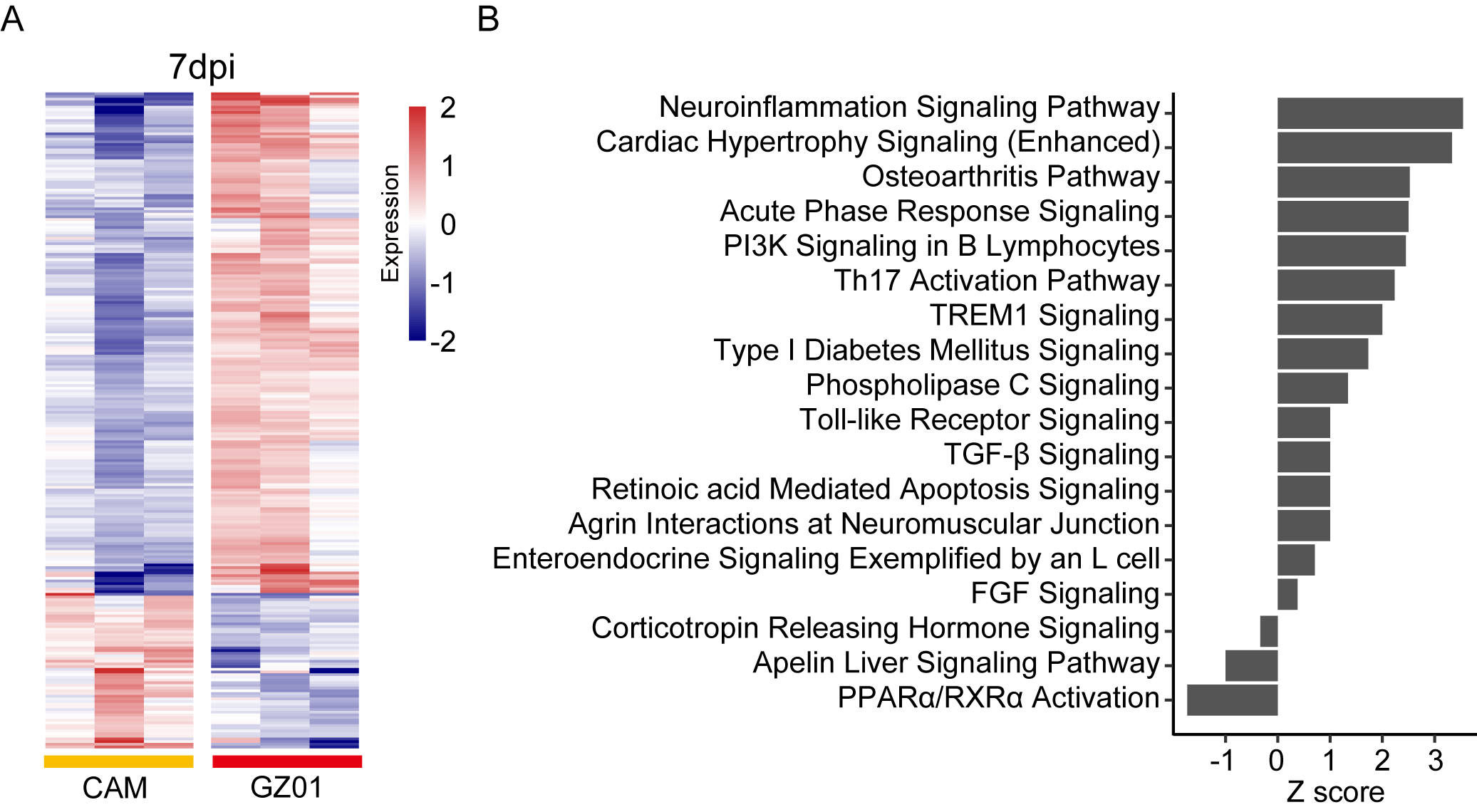

Supplement: Supplementary Figure 1 — (A), Heatmap of differentially expressed immune genes between CAM and GZ01 at 7 dpi. (B), Functional enrichment of differentially expressed genes between CAM and GZ01 at 7 dpi. [file Image_1.tif]

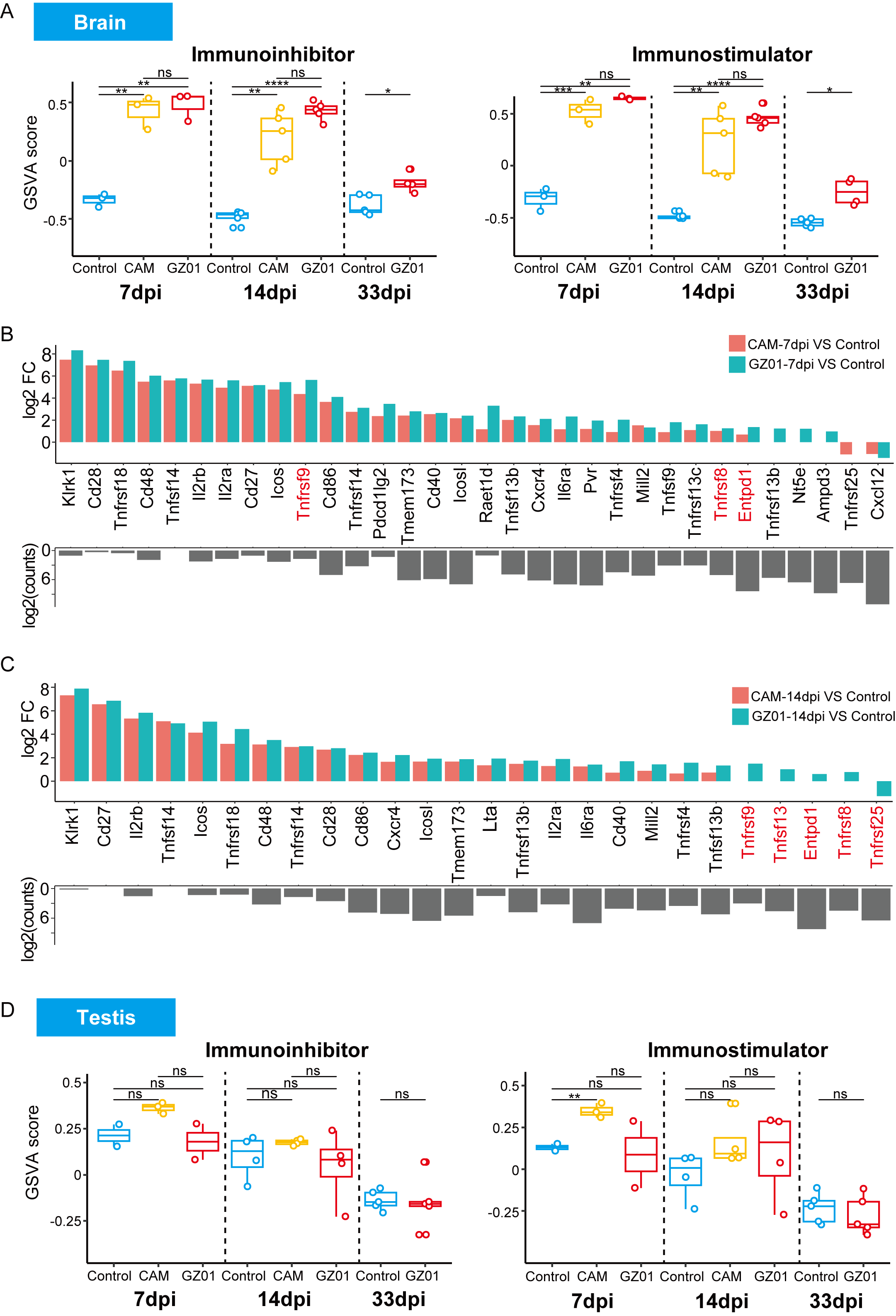

Supplement: Supplementary Figure 2 — Evaluation of immune checkpoint in tissues from mice infected by each strain. (A), Calculation of the immune checkpoint scores of mouse brain by GSVA. (B, C), Differential fold changes of immune stimulation gene from brain at 7 dpi and 14 dpi. (D), Calculation of immune checkpoint scores of mouse testes by GSVA. *P < 0.05; **P < 0.01, ***P < 0.001, ****P < 0.0001, ns, not significant (two-tailed unpaired Student’s t-test). [file Image_2.tif]

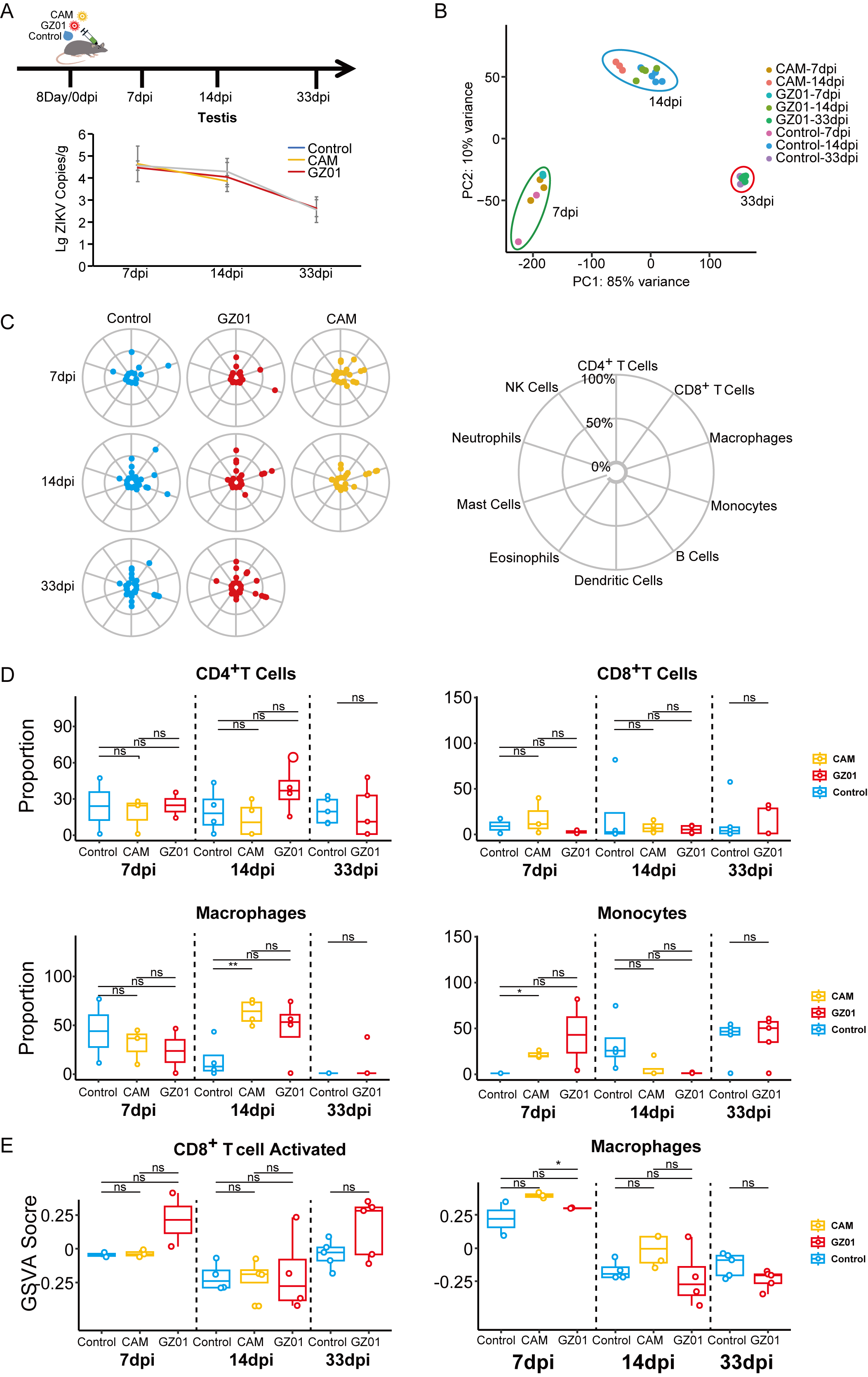

Supplement: Supplementary Figure 3 — Changes in the immune microenvironment of mice testes. (A), Viral loads of ZIKV in the mouse shown in line chart. (B), PCA displaying biological variation among different infection groups. (C), The proportion of ten main immune cell types. The immune cell ratio was calculated using expression data from the immuCC website. (D), Comparison of the immune cell ratio of immune cell subtypes in different infection groups. (E), Comparison of the enrichment scores of immune cell subtypes in different infection groups by GSVA. [file Image_3.tif]
